# The Microbiome of the Cosmopolitan Diatom *Leptocylindrus* Reveals Significant Spatial and Temporal Variability

**DOI:** 10.3389/fmicb.2018.02758

**Published:** 2018-11-15

**Authors:** Penelope A. Ajani, Tim Kahlke, Nachshon Siboni, Rick Carney, Shauna A. Murray, Justin R. Seymour

**Affiliations:** Climate Change Cluster, University of Technology Sydney, Sydney, NSW, Australia

**Keywords:** diatom, bacteria, phycosphere, network analysis, *Roseovarius*

## Abstract

The ecological interactions between phytoplankton and marine bacteria have important implications for the productivity and biogeochemistry of ocean ecosystems. In this study we characterized the microbial assemblages associated with multiple isolates of the ecologically important diatom *Leptocylindrus* using amplicon sequencing of the 16S rRNA gene, to examine levels of conservation of the microbiome across closely related species or strains. We also assessed if the microbiome structure of a given diatom strain was dependent on the location from which it was isolated and if the microbiome of cultured isolates significantly changed overtime from the seawater in which they were isolated. The bacterial assemblages from 36 strains belonging to three species (*Leptocylindrus* *danicus, Leptocylindrus convexus*, and *Leptocylindrus aporus*) isolated from six locations spanning > 1000 km of south east Australian coastline over 1 year, were dominated by the Rhodobacteraceae (∼60%) and the Flavobacteriaceae (∼10%). Across all strains, only one ‘core OTU’ (*Roseovarius* sp.) was identified across all samples. We observed no significant differences in bacterial community composition between diatom species. Significant differences in microbiome structure were, however, observed between diatom strains collected at different sampling times and from differing locations, albeit these two factors were coupled. Moreover, while bacterial communities under domestication varied from the seawater in which they were isolated, they remained specific to the location/month of origin, i.e., different regions and time points harbored distinct bacterial communities. Our study delivers new knowledge in relation to diatom-bacterial associations, revealing that the location/time from which a diatom is isolated plays an important role in shaping its microbiome.

## Introduction

The interactions between phytoplankton and bacteria are among the most significant ecological associations in the global ocean (Amin et al., 2012). While regularly considered in isolation, the ecology and physiology of these important functional groups of aquatic microorganisms are often closely coupled with interactions spanning mutualism, commensalism, parasitism, and competition (Seymour et al., 2017 and references therein). These dynamic relationships are often complex and multifaceted, with the, sometimes reciprocal, exchange of metabolites and info-chemicals regulating a suite of important ecosystem-level processes including nutrient transformation, primary production, toxin biosynthesis, and biogeochemical cycling (Amin et al., 2012, 2015; Durham et al., 2015). In addition to the recognition of the importance of phytoplankton-bacterial interactions within oceanographic and aquatic ecological contexts, efforts to disentangle the relationship between bacteri,a and phytoplankton is also gaining momentum in the field of biotechnology. The optimization of microalgal cultivation (Cho et al., 2015), the augmentation of high value bio-products (Richter et al., 2018), and their use in natural remediation processes (Gutierrez et al., 2013) are all examples where greater insights into microbial associations will benefit (Ramanan et al., 2016 and references therein).

Many species of marine phytoplankton exhibit strong ecological links with specific members of the Proteobacteria and Bacteriodetes, including species of *Sulfitobacter, Roseobacter, Alteromonas*, and *Flavobacterium* (Amin et al., 2012; Buchan et al., 2014). These bacterial associates have been demonstrated to modify the growth, behavior and physiology of the microalgal host (Sison-Mangus et al., 2014; Segev et al., 2016; Bolch et al., 2017; van Tol et al., 2017), and to become abundant during phytoplankton blooms, for which they may play a role in governing bloom dynamics (Buchan et al., 2014; Bunse et al., 2016; Hattenrath-Lehmann and Gobler, 2017; Needham et al., 2017; Song, 2017).

It is widely anticipated that many of these specific phytoplankton – bacterial interactions are based on the active exchange of signaling molecules and/or nutrients (Green et al., 2015; Seymour et al., 2017). While it was traditionally thought that these interactions would be simply based on the provision of the organic products of photosynthesis to bacteria and perhaps remineralized nutrients to phytoplankton (Azam and Ammerman, 1984; Legendre and Rassoulzadegan, 1995), it is becoming evident that the chemical exchanges between phytoplankton and bacteria are much more diverse, specialized, and complex. For instance, tryptophan/indole-3-acetic acid (IAA), a common and important hormone for plant growth and development, has been shown to be received by the diatom *Pseudo-nitzschia multiseries* from the bacteria *Sulfitobacter*, which subsequently promotes diatom cell division (Amin et al., 2015). Growth metabolites exuded from the diatom *Thalassiosira pseudonana* have been putatively linked to the growth of the bacteria *Ruegeria pomeroyi* (Durham et al., 2017). Furthermore, the bacterium *Mesorhizobium loti* has also been observed to supply vitamin B12 (cobalamin) to the freshwater green alga *Lobomonas*, which receives photosynthate from the phytoplankton cell in return (Grant et al., 2014).

Among the most widely studied phytoplankton-bacterial interactions are those between diatoms and bacteria. Diatoms are one of the most ecologically important phytoplankton groups in the global ocean (Malviya et al., 2016), carrying out 20% of the Earth’s photosynthesis (Armbrust, 2009) and dominating phytoplankton biomass in many coastal regions across the global ocean (Fawcett and Ward, 2011; Huang et al., 2011; Morales and Anabalon, 2012). The bacteria associated with diatoms often belong to distinct bacterial phylotypes (Alpha-, Beta,- and Gammaproteobacteria and Bacteriodetes) made up of a relatively few genera, such as *Roseobacter, Sulfitobacter*, and *Flavobacterium* (Amin et al., 2012) and their presence is often linked to specific positive and negative interactions with diatom cells. In return for diatom derived dissolved organic carbon, bacteria from these groups have been shown to provide diatom cells with vitamin B_12_, soluble iron (via specialized siderophores), and nitrogen (via certain nitrogen-fixing cyanobacteria) (Croft et al., 2005; Foster et al., 2011; Amin et al., 2012). In contrast, some bacteria from these groups produce algicides which may kill diatoms (Lau et al., 2007), an in turn some diatoms can produce antibacterial compounds as a defense feature (Desbois et al., 2009).

Most of our current knowledge of the interactions between diatoms and bacteria, however, is derived from examining cultured, model systems, which have focused on a limited number of algal species or strains, usually sourced from long-term culture collections (Amin et al., 2015; Moejes et al., 2017). Behringer et al., 2018 demonstrated that the bacterial communities associated with the diatom species *Nitzschia longissima* (three strains) and *Asterionellopsis glacialis* (four strains) displayed strong conservation across strains from the same species (conserved at the genus level), and that cultivation over time (>1 year) resulted in only small changes to the bacterial composition.

The genus *Leptocylindrus* is one of the most numerically dominant diatoms in the ocean (Malviya et al., 2016) and is a major component of the spring bloom period in southeastern Australia (Ajani et al., 2016b). With its ease of microscopic identification, single cell isolation and successful growth in culture, species belonging to this genus are ideal candidates for the examination of phytoplankton-bacterial interactions. To investigate the co-existence of natural communities of bacteria and *Leptocylindrus*, we addressed three questions: Do bacterial communities associated with closely related *Leptocylindrus* species differ between and within species? Does the bacterial community associated with *Leptocylindrus* significantly change over time (months) from the original seawater from which it was isolated? Is the microbiome of each diatom strain predetermined by its collection time or location? By characterizing the bacteria that live in association with marine phytoplankton, we may begin to understand the function of a host organism’s microbiome, and its interaction within this microenvironment.

## Materials and Methods

### Phytoplankton Collection, Culture Maintenance, and Species Characterization

**FIGURE 1:**
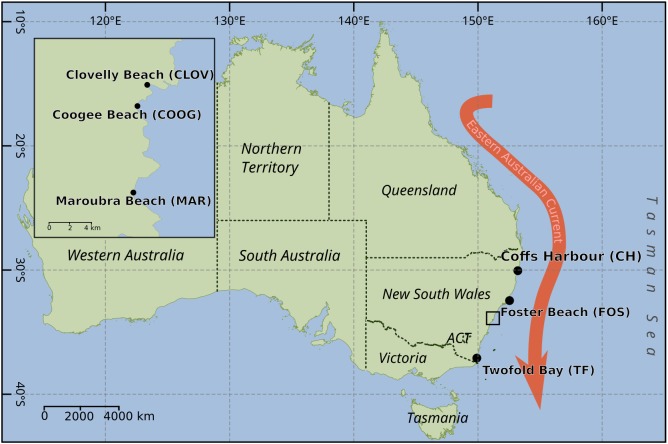
Map of Australia, showing the sampling locations for the isolation of the diatoms *Leptocylindrus* as shown by black dots. Black box indicates sampling locations within the urbanized area of Sydney. Red arrow indicated the dominant western boundary current along the southeast of Australia – the East Australian current.

Non-axenic monoclonal batch cultures of *Leptocylindrus* were established by single cell isolation using drawn out Pasteur pipettes (micropipettes) from net samples (20-μm mesh size) collected from six locations along the southeastern Australian coastline from December 2015 to October 2016. Sampling sites included Coffs Harbor (30.3°S, 153.1°E), Forster (32.2°S, 152.5°E), Coogee (33.9°S, 51.2°E), Clovelly (33.9°S, 151.26°E), Maroubra (33.9°S, 151.24°E) and Twofold Bay (37.08°S, 149.9°E) from north to south (Figure 1). The locations of Coogee, Clovelly and Maroubra are all located within the urban surrounds of the city of Sydney.

*Leptocylindrus* isolates were grown in 24-well plates containing autoclaved F/2 media (Guillard, 1975) and monitored using light microscopy. Once established, each strain was transferred to 50 mL culture flasks and maintained in a constant temperature incubator at 16–18°C under a white light flux of 60–100 μmol photon m^-2^ s^-1^ and a 12-h light (L):12-h dark (D) cycle. One mL of culture from each strain was transferred into fresh media every 2 weeks to maintain healthy and exponentially growing cultures.

Species identification was confirmed by both morphological examination, using transmission electron microscopy, and sequencing of the nuclear-encoded partial ITS1–5.8S–ITS2 region of rDNA using the primers ITS-for (5′-ATATGCTTAAATTCAGCGGGT-3′) and ITS-rev (5′-TTTCCGTAGCTGAACCTGCGG-3′) (see Ajani et al., 2016a for further details). At approximately the same time as diatom strain collection, bulk water samples (3 × 5 L) were collected from two near shore (1 m depth) sites at Maroubra Beach, (33.9°S, 151.24°E). From each of these bulk samples, 2.5 L was filtered through a 0.22 μm pore size membrane filter (Millipore) using a peristaltic pump. Filters were stored at -80°C prior to DNA extraction.

### DNA Extraction and 16S RNA Sequencing

To examine variability in the *Leptocylindrus* microbiome across strains, 5 mL of each strain was transferred to a 50 mL culture flask (× 3 replicates) at the final culture transfer and maintained as described above. For all strains, this occurred between 6 and 16 months after initial cell isolation (Supplementary Table 1). On day 18 (∼stationary phase) each replicate strain was harvested by centrifugation (2300 RCF for 5 min), and total DNA extracted from the pellet using a DNeasy^®^ PowerSoil^®^ Kit (Qiagen) as per manufacturer’s protocol. Bulk water samples were filtered through 0.22 μm pore-size membrane filters (Millipore) using a peristaltic pump and stored at -80°C prior to DNA extraction. For water samples, DNA extraction was performed using the Powerwater DNA isolation Kit (Qiagen) in accordance with the manufacturer’s instructions. DNA quantity and quality was examined spectroscopically with a Nanodrop (ND-1000, Thermo Scientific, Woltham, MA, United States).

The V1-V3 variable region of the bacterial 16S rRNA gene was amplified by PCR, using the primers 27F (AGAGTTTGATCMTGGCTCAG, Lane, 1991) and 519R (GWATTACCGCGGCKGCTG, Turner et al., 1999). 16S rRNA amplicon sequencing was subsequently performed on the Illumina MiSeq platform (Molecular Research LP; Shallowater, TX, United States) following the manufacturer’s guidelines. Raw data files in FASTQ format were deposited in NCBI Sequence Read Archive (SRA), with the study accession numbers PRJNA488135 and SRP143624 (diatom and bulk water samples, respectively).

### Sequence Data Processing and Bacterial Community Analyses

Bacterial 16S rRNA sequencing data was processed using a customized bioinformatic workflow as described in Kahlke (2018); Ampli-tools (Version 1.0); Zenodo]. Briefly, paired-end 16S sequences were joined using FLASH (Magoc and Salzberg, 2011) and subsequently trimmed using Mothur (Schloss et al., 2009) (maxhomop = 6, maxambig = 0, minlength = 434, maxlength = 489). The resulting fragments were clustered into operational taxonomic units (OTUs) at 97% identity and chimeric sequences were identified using vsearch (Rognes et al., 2016) and the Silva (v128) database. Taxonomy was assigned using QIIME (Caporaso et al., 2010) and BLAST against the Silva v128 database. Sequences were rarefied to 15,000 fragments to limit the effects of uneven sequencing depth.

Bacterial community patterns were examined for differences among diatom strains and species, date of isolation (month) and sampling location. Alpha diversity parameters (Shannon Diversity Index, Chao1, and the number of observed OTUs per sample) of the rarefied sequences were calculated in QIIME (Caporaso et al., 2010). These were compared using Kruskal–Wallis (non-parametric one-way analysis of variance by ranks, *p* < 0.05) in STATISTICA v13.0 (StatSoft, Tulsa, OK, United States) to test for differences between time of isolation (months 1, 2, 10, and 12) and between sampling locations. A Jackknife (Qiime v1.9) comparison of the weighted rarefied data was then used to examine to what extent the bacterial community differed between the original seawater at the time of collection.

Non-parametric (multivariate) analyses were also undertaken to compare *Leptocylindrus* microbiomes between strains (and replicates), species, months and sampling locations. Using the relative abundance data (after a log [x+1] transformation to ‘down weight’ the most abundant bacterial OTUs) multidimensional scaling was applied to provide an ordination of the data using the Bray–Curtis similarity measure using PRIMER-E v6 (Clarke and Gorley, 2006). Analysis of similarity (ANOSIM) was then used to investigate differences, and where significant, the similarity percentages procedure (SIMPER) was used to identify the OTUs most responsible for these discriminations (Clarke and Warwick, 2001).

### *Leptocylindrus* Core Microbiome

To identify OTUs of putative ecological importance to *Leptocylindrus*, the core microbiome was determined for: (a) all strains to identify the OTUs that were universally associated with the three *Leptocylindrus* species, and (b) strains taken from the same location to investigate the effect of location on core microbiome structure. An OTU was defined as a member of the core microbiome if it was present in at least two out of three replicates for each sample type (i.e., allowing for one replicate outlier per group). Similarly, for the location-specific core microbiome, an OTU was considered a member of the core microbiome if it was present in all replicates of all strains taken at the same location. This more stringent cut off was chosen due to a lower number of strains and the assumption that geographically closer sampling sites have a higher chance of sharing common OTUs.

### Bipartite Network Analysis

To further compare the composition of bacterial OTU assemblages across locations/months, a bipartite network was created using 16S rRNA amplicon data. The python command make_bipartite_network.py part of QIIME v1.9 was used for this method as previously described by Muegge et al. (2011) and Gibbons et al. (2013). This produced a dataset consisting of two node types: strains grouped by location/month of isolation and the abundance of bacterial OTUs, that is, the number of sequence reads detected for each OTU at each respective location/month. A bipartite network graph was produced from this new dataset by first pooling strain replicates, and then using the attribute circle layout feature in Cytoscape 3.6.0 (Shannon et al., 2003). This allowed the bacterial OTUs that were unique to only one location or month to be distinguished from those that were common across two or more location or months.

## Results

### Strain Characterization

A total of 36 strains of the diatom genus *Leptocylindrus* were collected from six locations spanning 1069 km of coastline from December 2015 to October 2016 (<1 year). Using the nuclear-encoded partial ITS1–5.8S–ITS2 region of rDNA, the isolated strains were found to span three species, namely: *Leptocylindrus* *danicus, Leptocylindrus convexus*, and *Leptocylindrus aporus*, with 31, 4 and 1 strains of each species established, respectively (Supplementary Table 1).

A total of 107 samples (3 × 36 strains; with the exception of strain FOS180216-21, for which only 2 of 3 replicates yielded enough DNA for downstream processing) were analyzed for their bacterial community composition. Comparative sequence analysis of the 16S rRNA gene libraries revealed the total number of raw reads across all replicates ranged from 91,360 to 214,451 reads and from 23,519 to 173,004 reads after quality filtering (Supplementary Table 1). Analysis of the normalized (15,000 rarefaction) sequences from replicates revealed a total of 1956 individual OTUs across all samples.

### *Leptocylindrus* Strain Comparisons

The Shannon Diversity Index for the microbiome associated with each strain ranged from 1.97 (*L. danicus* from Twofold Bay, strain TF251016-1) to 4.83 (*L. danicus* from Coffs Harbor, strain CH230116-7). Using the Chao1 estimator, the lowest diversity was again within the *L. danicus* strain isolated from Twofold Bay (strain TF251016-5), whilst the greatest species richness was observed in *L. aporus* from Coogee (strain COOG100215-7). The greatest overall number of rarefied OTUs (289) was recorded from a strain of *L. danicus* from Forster (strain FOS180216-2), whilst the lowest (66) was observed from *L. danicus* isolated from Twofold Bay (TF251016-5).

**FIGURE 2:**
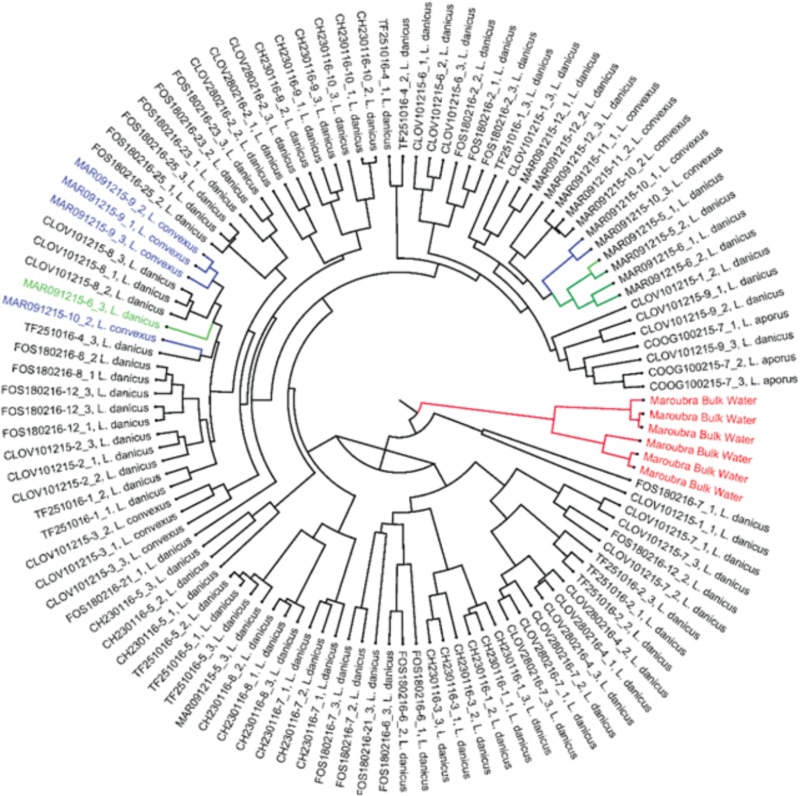
Jack knife phylogeny based on the weighted rarefied data shows Maroubra bulk water samples (shown in red) clearly separated from other bacterial communities associated with the diatom *Leptocylindrus* isolated from the same location. Green: bacterial communities associated with *Leptocylindrus danicus*; blue: bacterial communities associated with *Leptocylindrus convexus*.

Significant differences in bacterial diversity were observed between time of strain isolation (species pooled) for all diversity indices measured (eg., Shannon *p* < 0.001) and similarly, when only *L. danicus* strains were examined (*p* < 0.001). When strains were pooled for species and months, a significant difference between location of strain isolation was also observed (*p* < 0.001) and was similarly observed when only *L. danicus* strains were examined (*p* = 0.001) (Supplementary Tables 2, 3). Using the Jackknife resampling technique, the bacterial community in the seawater around the time of collection clearly discriminated from all other cultured strains (Figure 2).

**FIGURE 3:**
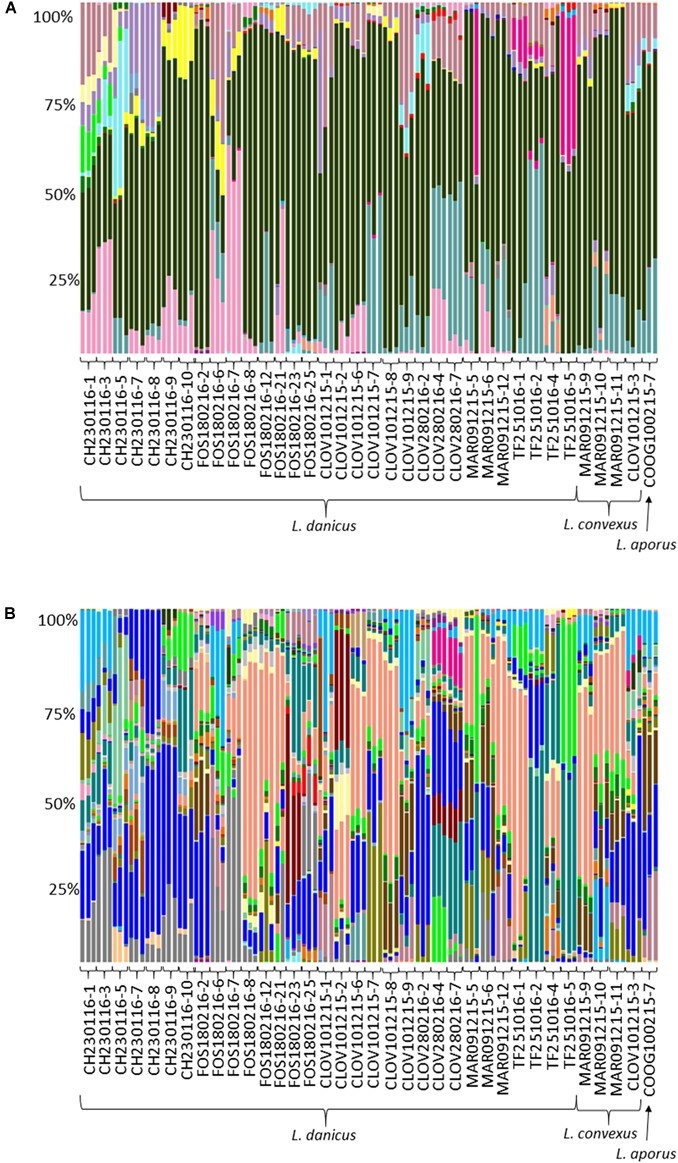
Comparative sequence analysis of normalized (15000 rarefaction) 16S ribosomal RNA (rRNA) gene libraries from all diatom strains. Each strain has three replicates with the exception of strain FOS180216-21, for which only 2 of 3 replicates yielded enough DNA for downstream processing. **(A)** Relative percent abundance at OTU level; ∼58% were made up of *Roseovarius* 18% (coral); Rhodobacteraceae; Ambiguous taxa 17.1% (bright blue); Rhodobacteraceae, uncultured sp. 7% (aqua); *Owenweeksia* 5.5% (gray); *Haliea* 5% (light blue); *Donghicola* 4.6% (brown); and **(B)** relative percent abundance at family level; ∼95% of all sequences were made up of eight families Rhodobacteraceae 60.6% (black); Flavobacteriaceae 10.4% (turquoise); Halieaceae 7.3% (dark pink); Cryomorphaceae 6.7% (pale pink); Alteromonadaceae 3.9% (purple); Erythrobacteraceae 2% (yellow); Pseudoalteromonadaceae 2% (bright pink); and Sphingomonadaceae 1.9% (light blue).

Of all classified OTUs, 81.8% were Proteobacteria and 17.8% Bacteroidetes, with very minor contributions from the Actinobacteria, Cyanobacteria, Planctomycetes and Verrucomicrobia (total 0.4%) (Supplementary Table 4 and Figure 3A). At the family level, the most commonly observed family were the Rhodobacteraceae (60.6%), followed by the Flavobacteriaceae (10.4%), Halieaceae (7.3%), Cryomorphaceae (6.7%), Alteromonadaceae (3.9%), Erythrobacteraceae (2%), Pseudoalteromonadaceae (2%), and the Sphingomonadaceae (1.9%) (Figure 3B). All other contributions at this taxonomic level comprised < 1% of the community (Figure 3B). Both the most frequently occurring (present in 106/107 replicate samples), and the most relative abundant OTU (18.5%), across all strains was an OTU (7767), classified as an uncultured *Roseovarius* sp from within the Rhodobacteraceae (Supplementary Table 4 and Figure 3A).

**FIGURE 4:**
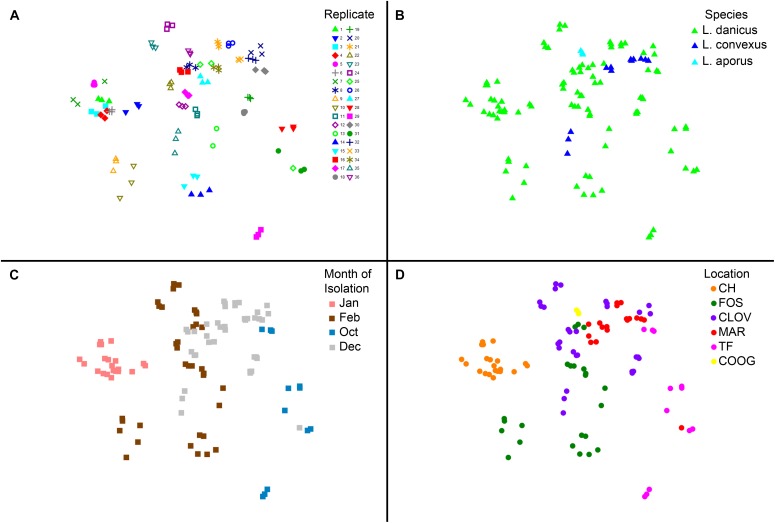
**(A–D)** Multivariate two dimensional scatter plots plots showing the variation in bacterial communities between **(A)** replicates (each strain contains three replicates); **(B)** between species of the diatom *Leptocylindrus* – *L. danicus, L. convexus*, and *L. aporus*; C. between sampling times (4 months) and **(D)** between collection locations along the east coast of Australia.

No significant differences in diatom microbiome structure were apparent between replicates of a given strain or between species (*L. danicus, L. convexus*, R statistic = -0.079, *p* = 0.844; *L. danicus, L. aporus* *R* = -0.177, *p* = 0.864; *L convexus, L. aporus* *R* = 0.161, *p* = 0.119) (Figure 4A). However, consistent with the patterns in alpha-diversity, significant differences were observed between the microbiomes of *Leptocylindrus* strains collected across different sampling months (global *R* = 0.635, *p* = 0.001) (Figure 4B). Significant spatial variability was also seen among strains collected from differing locations, when species and sampling times were pooled (global *R* = 0.573, *p* = 0.001) (Figure 4C), with the microbiomes of *Leptocylindrus* strains collected from Sydney urban sites (Clovelly, Maroubra, *R* = 0.018, *p* = 0.3; Clovelly, Coogee *R* = -0.22, *p* = 0.95, Maroubra, Coogee, *R* = 0.431, *p* = 0.018) clustering together and clearly discriminated from those strains collected from the northern-most and southern-most sites at Coffs Harbor and Twofold Bay (Figure 4D). When the analysis was repeated using only *L. danicus* strains, the same pattern was observed (data not presented). Pairwise SIMPER analyses revealed between 4 to 8 bacterial OTUs explained > 5% of the cumulative contribution to dissimilarity among the microbiome of *Leptocylindrus* strains collected at differing sampling locations (Sydney sites combined), with OTUs classified as Rhodobacteraceae (*Roseovarius* and *Donghicola*) more abundant in Sydney compared to strains collected from other locations, and OTUs classified as Cryomorphaceae (*Owenweeksia*) more abundant in *Leptocylindrus* strains from Coffs Harbor compared to those isolated from other locations (Supplementary Table 5).

### *Leptocylindrus* Core Microbiome

To understand the OTUs of putative ecological importance to *Leptocylindrus*, we determined the “core microbiome” across all strains cultivated. The only core OTU present across all strains/replicates was OTU 7767, *Roseovarius* sp., which was also the most abundant OTU across all strains (see above). When the core microbiome was calculated according to location of strain isolation, eleven core OTUs were identified at Coffs Harbor (73% belonging to the family Rhodobacteraceae); 4 at Forster (all Rhodobacteraceae, including one *Roseovarius*); 4 at Clovelly (all Rhodobacteraceae, including two *Roseovarius*); 156 at Coogee (77% Rhodobacteraceae); 10 at Maroubra (all Rhodobacteraceae, including several *Pseudoroseovarius* and one *Roseovarius* OTUs); and 3 at Twofold Bay (all Rhodobacteraceae, including one *Roseovarius* and one *Pseudoroseovarius*) (Supplementary Table 6). In addition to the most abundant bacterium observed across all strains (Rhodobacteraceae, *Roseovarius* sp. uncultured bacterium OTU 7767, see above), another unidentified Rhodobacteraceae (OTU 9240) was present in the core microbiome of strains from all locations with the exception of Coffs Harbor (Supplementary Table 6). Other OTUs, Rhodobacteraceae *Shimia* (OTU 10067); Rhodobacteraceae, *Pseudoroseovarius*, uncultured alpha proteobacteria (OTUs 10098, 10103, and 10702); and unidentified Rhodobacteraceae (OTU 10977), were only observed in the core microbiomes of strains from Coogee and Maroubra (Supplementary Table 6).

### Bipartite Network Analysis

**FIGURE 5:**
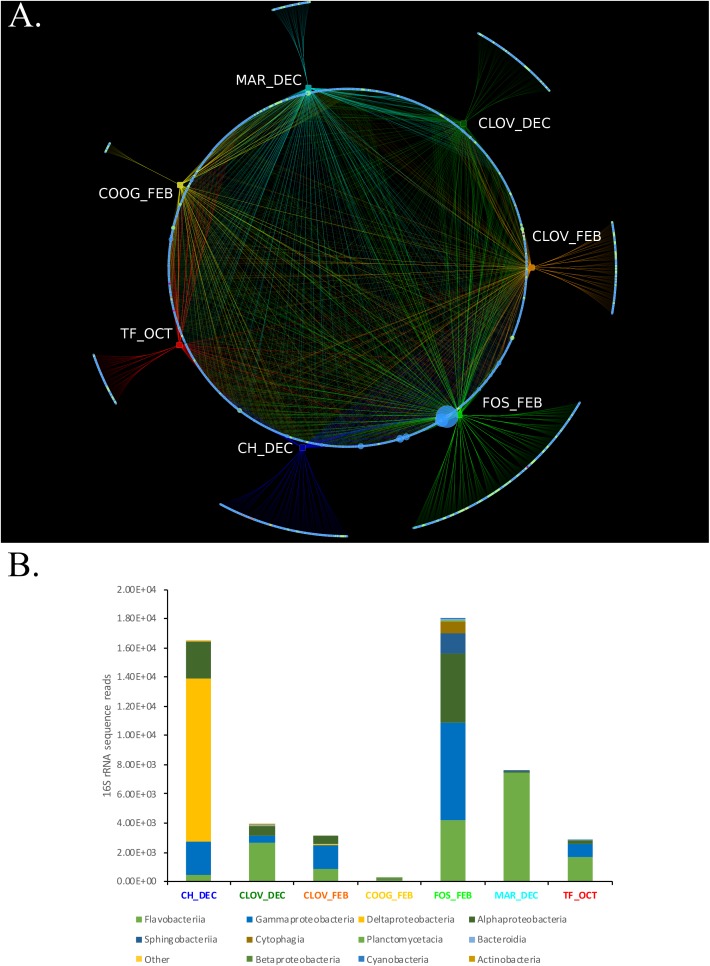
**(A,B)** A Phytoplankton isolates harbor different bacterial consortium depending on the location and time of sampling from the environment. **(A)** Bipartite analysis of 7 location/timepoints (rectangles) and 1957 bacterial OTU nodes (circles), yielded 4605 edges (lines), each representing the sum number of 16S rRNA sequence reads for each bacterial OTU at their respective location/timepoint. Nodes representing bacterial OTUs were colored by phyla assignment; Actinobacteria (yellow), Bacteroidetes (light green), Cyanobacteria (teal), Planktomycetes (purple), Verrucomicrobia (violet), and Proteobacteria (blue). Edge colors were set to match the color of their respective location/time point nodes. Bipartite analysis revealed 1,055 “common” bacterial OTUs that were connected via an edge with > 2 location/time point nodes. Whereas, 902 bacterial OTUs were unique to only one location/timepoint, including CH_DEC (188), CLOVE_DEC (131), CLOV_FEB (131), COOG_FEB (15), FOS_FEB (305), MAR_DEC (55), and TF_OCT (77). **(B)** Stacked bar plots showing the phylogenetic composition and overall abundance of unique OTUs at each location/time point.

Bipartite analysis of the strain by location/month of isolation (7) and the abundance of bacterial OTUs (total OTUs identified 1956) identified 1,055 OTUs that were common across > 2 location/month(s) nodes, while 902 OTUs were unique to only one location/month node (Figure 5A). The number of unique OTUs for each location/month node ranged from 15 (Coogee, February) to 305 (Forster, February), belonging to six major phyla: Actinobacteria, Bacteroidetes, Cyanobacteria, Planktomycetes, Verrucomicrobia, and Proteobacteria (Figure 5B).

## Discussion

Identifying the key bacteria associated with ecologically significant diatoms is a vital precursor to understanding the intricate relationships between these two microbial groups in the global ocean. While previous algal-bacterial studies have examined a limited number of model organisms from long term laboratory culture collections, our study examines 16S rRNA sequences obtained from 36 clonal isolates spanning 3 species of the diatom *Leptocylindrus*, collected over a 1-year period and from six locations spanning 1000 km of the coastline. Data collected over the past 50 years from a long-term coastal monitoring station (Port Hacking – at a depth 100 m) has revealed that *Leptocylindrus* is one of the most abundant genera observed in these coastal waters, dominating the spring and summer diatom bloom periods (Ajani et al., 2014a,b). The ecological importance of *Leptocylindrus* has also been recognized by the recent Tara Ocean expeditions, reporting this genus to be one of the five most abundant diatoms globally (Malviya et al., 2016). Moreover, *Leptocylindrus* is reported as one of the least diverse genera, making it an ideal candidate to investigate the mechanisms underlying bacterial-diatom interactions.

### *Leptocylindrus* Strain and Species Comparisons

Our results indicate that the bacterial communities associated with different strains of *Leptocylindrus* recently isolated from natural environments were generally conserved across three species, namely *L. danicus, L. convexus, and L. aporus*. In all *Leptocylindrus* strains the bacterial community was dominated by the Proteobacteria (∼80%) and the Bacteroidetes (∼20%), while at the family level the most important bacterial groups were the Rhodobacteraceae (∼60%) and Flavobacteriaceae (∼10%). This is consistent with previous studies that have demonstrated that these are the dominant heterotrophic bacterial groups associated with diatoms (Amin et al., 2012 and references therein) and other phytoplankton (Buchan et al., 2014).

More specifically, the most abundant bacterial group within *Leptocylindrus* microbiomes, the *Roseobacter*, are one of the most pervasive microbial genera interacting with phytoplankton in the marine ecosystem (Moran et al., 2003; Ramanan et al., 2016). The second dominant group, the Flavobacteria, are abundant in coastal upwelling regions from temperate to polar oceans and are also widely recognized as phytoplankton-associates (Buchan et al., 2014). Of the 10–20% of Bacteroidetes that are present in the open ocean bacterial community, the Flavobacteria are the most common. They are also commonly reported during phytoplankton blooms, where distinct phylotype succession patterns have been observed (Buchan et al., 2014 and references therein). Thought to be more opportunistic than the Roseobacter clade, this group has been reported to more frequently be particle-/diatom-attached rather than free-living microbes (Grossart et al., 2005). It is hypothesized that this niche separation is one way in which competing species of bacteria (Rosebacter and Flavobacter) can partition their influence on the ecophysiology of diatoms and successfully coexist in the marine environment (Teeling et al., 2016).

While our study showed bacterial assemblages were generally conserved across closely related species, differing diatom genera eg., *Ditylum, Thalassiosira, Astrionella, Chaetoceros, Leptocylindrus* and *Coscinodiscus*, have been shown to develop and maintain distinct bacterial satellite assemblages even when ‘challenged’ with alternate bacterial assemblages (Schafer et al., 2002). A recent study by Behringer et al. (2018) also supported this conservation of bacterial communities at the genus level, with strains of the diatoms *A. glacialis* and *N. longissima* maintaining stable microbial communities across strains and highly conserved microbiomes at the phytoplankton genus level. In the same way, the bacterial communities associated with strains of *Pseudo-nitzsc*hia spanning six different species were found to be similar, with significant microbiome differences only reported between toxigenic and non-toxigenic species (Guannel et al., 2011). Whilst both the toxic species (*P. multiseries*) and the non-toxic species (*P. delicatissima*) hosted common bacterial members (eg., Roseobacter, Gammaproteobacteria and Flavobacteria), the bacterial community of the toxigenic species (*P. multiseries*) was far less abundant and diverse than the non-toxigenic species. Furthermore, when two axenic marine diatoms (*Thalassiosira rotula* and *Skeletonema costatum*) were inoculated with natural bacterial assemblages, the resultant free-living and attached bacteria differed both between the diatom species and between the growth stages within a single species (Grossart et al., 2005). Of the two distinct bacterial communities that evolved over time under cultivation, members of the Roseobacter group dominated the free-living community while the Flavobacteria-Sphingobacteria were the main representatives in the diatom-associated assemblage.

### *Leptocylindrus* Spatial and Temporal Comparisons

Bacterial communities co-existing with certain species of diatoms are generally dominated by a distinct consortium of bacteria that are conserved over geographical locations and time under laboratory cultivation (Guannel et al., 2011). Amin et al. (2012) reported consistent associations between specific clades of bacteria associated with strains of *Pseudo-nitzschia* isolated from the Atlantic Ocean and the North Pacific Ocean. Whilst these conserved consortia may exist at the genus level or above, our data suggests however, that at the operational taxonomic unit (OTU) level, significant spatial and temporal variability is retained under cultivation across diatom microbiomes. Specifically, we observed significant dissimilarity in the composition of the *Leptocylindrus* microbiome (at the OTU level) between *Leptocylindrus* strains from differing locations along the east coast of Australia, with a higher diversity and more unique OTUs associated with *Leptocylindrus* strains isolated from the more northern locations compared to those from the south. Furthermore, our data also revealed a Sydney “urban” cluster, where bacterial communities within *Leptocylindrus* strains originating from sites within this region (Coogee, Clovelly, and Maroubra) differed from strains collected in the more northern sampling locations at Coffs Harbor and Forster, and from the more southern location at Twofold Bay. These differences were attributed to a greater abundance of the Rhodobacteraceae (*Roseovarius* and *Donghicola*) within the *Leptocylindrus* microbiome in strains originating in the Sydney region compared to other locations, whilst the Cryomorphaceae (*Owenweeksia*) were more abundant members of the *Leptocylindrus* microbiome in strains originating from Coffs Harbor compared to other locations. While our experimental design is not balanced, that is, it is not easy to disentangle the effect of location and sampling time on the bacterial communities associated with *Leptocylindrus* strains, our data suggests that different regions and time points harbor distinct bacterial communities. This may be explained by the fact that different members of the same functional guild may share similar metabolic functions and may therefore populate the same ecological niche in different regions. However, future studies focusing on the conservation of key metabolic elements of these bacteria are needed to support this hypothesis.

While it was outside the scope of our study to examine the evolution of bacterial communities under cultivation over time, Behringer et al. (2018) clearly demonstrated that the microbiome associated with multiple strains of two diatom species remained stable over time. Bacterial communities re-sampled over approximately 1 year (at 20, 200, and 400 days) displayed only small changes in composition. One caveat to their approach, however, was that their diatom microbiomes were characterized 20 days after initial isolation, during which time there may have been changes to the assemblage from the seawater from which they were originally isolated. Our study extends this work by showing that diatom microbiomes do indeed vary from the seawater in which they were isolated yet remain specific to their location of origin under domestication. Moreover, we suggest that a more intensive temporal sampling campaign, including a fully nested factorial experimental design and an examination of the original seawater from which all diatoms are isolated, would further close the gap in our knowledge surrounding the multiscaled, temporal variability of diatom microbiomes from natural assemblages.

### *Leptocylindrus* Core Microbiome

In order to understand the function of a host organism’s microbiome, which may play a critical role in its interaction with the environment, many researchers are beginning to investigating the ‘core microbiome’ of specific marine taxa (Schmitt et al., 2012; Ainsworth et al., 2015; Bengtsson et al., 2017; Lawson et al., 2018). Examination of the core microbiome of *Leptocylindrus* identified only one core OTU, *Roseovarius* sp. Species belong to *Roseovarius* are the most predominant microbial taxa documented in the marine algal-bacteria literature (Rappe et al., 2000; Grossart et al., 2005; Guannel et al., 2011; Amin et al., 2012; Ramanan et al., 2016 and references therein). Unsurprisingly, it was the family Rhodobacteraceae (to which *Roseovarius* sp. belongs) which also characterized ∼70–100% of the core microbiomes belonging to each location. Interestingly however, network analysis revealed that the microbiome across all strains were both spatially and temporally variable, that is, *Leptocylindrus* strains collected from specific locations and months harbored a unique set of bacterial OTUs (as well as many shared OTUs). A very likely explanation for this variability may be that the local environment (light penetration, water temperature and nutrient availability) may drive community composition.

## Conclusion

The diatom genus *Leptocylindrus* is a dominant component of the phytoplankton community, particularly in coastal upwelling systems around the world. Examination the microbiome of 36 cultivated strains belonging to three species belonging to this genus revealed Roseobacter and Flavobacteria to be the dominant taxa across all species and strains, with a single-taxa core microbiome determined. When the bacterial community was examined across multiple locations and sampling times however, significant spatial and temporal variability amongst strains was observed. Furthermore, while communities under domestication varied from the seawater in which they were isolated, they remained specific to the location/month of origin, that is, different regions and time points harbored distinct bacterial communities. Until now our knowledge of diatom microbiomes has been limited to a few strains/species which have often been sourced from long-term culture collections, with little information or examination of the environment from where they were isolated. Our study delivers new knowledge concerning diatom-bacterial associations, by revealing that the location from which a diatom is isolated plays an important role in shaping its microbiome. A major challenge will be to elucidate the metabolic nature of the relationship between these ecologically relevant partners that incorporates this temporal and spatial variability.

## Author Contributions

PA, SM, and JS are responsible for the conceptualization of this work. PA, TK, NS, and RC performed the analysis. All authors are responsible for the writing, reviewing, and editing of this manuscript.

## Conflict of Interest Statement

The authors declare that the research was conducted in the absence of any commercial or financial relationships that could be construed as a potential conflict of interest.
