# Supplementary material for: The Microbiome of the Cosmopolitan Diatom Leptocylindrus Reveals Significant Spatial and Temporal Variability
Source: Front Microbiol. 2018 Nov 15;9:2758. doi: 10.3389/fmicb.2018.02758 (PMC6249420; doi:10.3389/fmicb.2018.02758)
Supplement: Supplementary file 1 [file Table_1.docx]

Supplementary Table 1. *Leptocylindrus* strains established for microbiome analysis from sampling locations north to south along east coast of Australia; ^#^sequences from Ajani et al. 2016.

| **Location** | **Strain/Rep Designation** | **Date of Collection** | ***Leptocylindrus***  **Species** | **Genbank Accession No.** | **Total Raw Reads** | **Total Fragments** |
| --- | --- | --- | --- | --- | --- | --- |
|  |  |  |  |  |  |  |
| **Coffs** | CH230116-1_1 | 23 Jan 2016 | *L. danicus* | MH722316 | 129448 | 76030 |
| **Harbour** | CH230116-1_2 |  | *L. danicus* |  | 163193 | 94273 |
|  | CH230116-1_3 |  | *L. danicus* |  | 134839 | 84046 |
|  | CH230116-3_1 | 23 Jan 2016 | *L. danicus* | KY008547^#^ | 143903 | 85129 |
|  | CH230116-3_2 |  | *L. danicus* |  | 154256 | 37793 |
|  | CH230116-3_3 |  | *L. danicus* |  | 148460 | 92829 |
|  | CH230116-5_1 | 23 Jan 2016 | *L. danicus* | KY008560^#^ | 169753 | 119482 |
|  | CH230116-5_2 |  | *L. danicus* |  | 170282 | 109026 |
|  | CH230116-5_3 |  | *L. danicus* |  | 159013 | 107131 |
|  | CH230116-7_1 | 23 Jan 2016 | *L. danicus* | MH722317 | 160044 | 85004 |
|  | CH230116-7_2 |  | *L. danicus* |  | 161632 | 98783 |
|  | CH230116-7_3 |  | *L. danicus* |  | 188106 | 77449 |
|  | CH230116-8_1 | 23 Jan 2016 | *L. danicus* | MH722318 | 134286 | 100321 |
|  | CH230116-8_2 |  | *L. danicus* |  | 150836 | 112148 |
|  | CH230116-8_3 |  | *L. danicus* |  | 185112 | 140159 |
|  | CH230116-9_1 | 23 Jan 2016 | *L. danicus* | KY008550^#^ | 192919 | 137562 |
|  | CH230116-9_2 |  | *L. danicus* |  | 176178 | 122469 |
|  | CH230116-9_3 |  | *L. danicus* |  | 214451 | 148406 |
|  | CH230116-10_1 | 23 Jan 2016 | *L. danicus* | KY008548^#^ | 176743 | 100709 |
|  | CH230116-10_2 |  | *L. danicus* |  | 197871 | 110896 |
|  | CH230116-10_3 |  | *L. danicus* |  | 209483 | 111559 |
| **Forster** | FOS180216-2_1 | 18 Feb 2016 | *L. danicus* | MH722319 | 109059 | 23519 |
|  | FOS180216-2_2 |  | *L. danicus* |  | 130841 | 43476 |
|  | FOS180216-2_3 |  | *L. danicus* |  | 162030 | 36866 |
|  | FOS180216-6_1 | 18 Feb 2016 | *L. danicus* | MH722320 | 155764 | 81093 |
|  | FOS180216-6_2 |  | *L. danicus* |  | 145201 | 80090 |
|  | FOS180216-6_3 |  | *L. danicus* |  | 150140 | 69137 |
|  | FOS180216-7_1 | 18 Feb 2016 | *L. danicus* | KY008545^#^ | 149579 | 68075 |
|  | FOS180216-7_2 |  | *L. danicus* |  | 143855 | 61097 |
|  | FOS180216-7_3 |  | *L. danicus* |  | 151267 | 64203 |
|  | FOS180216-8_1 | 18 Feb 2016 | *L. danicus* | *auxopore | 120819 | 27475 |
|  | FOS180216-8_2 |  | *L. danicus* |  | 143058 | 55030 |
|  | FOS180216-8_3 |  | *L. danicus* |  | 146693 | 49192 |
|  | FOS180216-12_1 | 18 Feb 2016 | *L. danicus* | MH722321 | 168488 | 131573 |
|  | FOS180216-12_2 |  | *L. danicus* |  | 185250 | 149711 |
|  | FOS180216-12_3 |  | *L. danicus* |  | 162303 | 130968 |
|  | FOS180216-21_1 | 18 Feb 2016 | *L. danicus* | MH722322 | 124925 | 92611 |
|  | FOS180216-21_3 |  | *L. danicus* |  | 152266 | 117691 |
|  | FOS180216-23_1 | 18 Feb 2016 | *L. danicus* | MH722323 | 161228 | 87423 |
|  | FOS180216-23_2 |  | *L. danicus* |  | 164543 | 78232 |
|  | FOS180216-23_3 |  | *L. danicus* |  | 138403 | 71307 |
|  | FOS180216-25_1 | 18 Feb 2016 | *L. danicus* | KY008568^#^ | 131844 | 35968 |
|  | FOS180216-25_2 |  | *L. danicus* |  | 101059 | 28917 |
|  | FOS180216-25_3 |  | *L. danicus* |  | 130981 | 39327 |
| **Clovelly** | CLOV101215-1_1 | 10 Dec 2015 | *L. danicus* | KY008538^#^ | 129606 | 41092 |
|  | CLOV101215-1_2 |  | *L. danicus* |  | 154447 | 107461 |
|  | CLOV101215-1_3 |  | *L. danicus* |  | 151060 | 109687 |
|  | CLOV101215-2_1 | 10 Dec 2015 | *L. danicus* | KY008536^#^ | 152108 | 106216 |
|  | CLOV101215-2_2 |  | *L. danicus* |  | 169590 | 110001 |
|  | CLOV101215-2_3 |  | *L. danicus* |  | 166853 | 107696 |
|  | CLOV101215-3_1 | 10 Dec 2015 | *L. convexus* | KY008563^#^ | 135585 | 83175 |
|  | CLOV101215-3_2 |  | *L. convexus* |  | 147313 | 77933 |
|  | CLOV101215-3_3 |  | *L. convexus* |  | 152887 | 84159 |
|  | CLOV101215-6_1 | 10 Dec 2015 | *L. danicus* | KY008551^#^ | 152558 | 84928 |
|  | CLOV101215-6_2 |  | *L. danicus* |  | 149875 | 68106 |
|  | CLOV101215-6_3 |  | *L. danicus* |  | 91360 | 47592 |
|  | CLOV101215-7_1 | 10 Dec 2015 | *L. danicus* | KY008554^#^ | 141943 | 76017 |
|  | CLOV101215-7_2 |  | *L. danicus* |  | 157930 | 79029 |
|  | CLOV101215-7_3 |  | *L. danicus* |  | 181350 | 95430 |
|  | CLOV101215-8_1 | 10 Dec 2015 | *L. danicus* | MH722324 | 169314 | 143576 |
|  | CLOV101215-8_2 |  | *L. danicus* |  | 201877 | 157745 |
|  | CLOV101215-8_3 |  | *L. danicus* |  | 169909 | 132954 |
|  | CLOV101215-9_1 | 10 Dec 2015 | *L. danicus* | KY008537^#^ | 132619 | 89880 |
|  | CLOV101215-9_2 |  | *L. danicus* |  | 143252 | 95910 |
|  | CLOV101215-9_3 |  | *L. danicus* |  | 154268 | 103766 |
|  | CLOV280216-2_1 | 28 Feb 2016 | *L. danicus* | MH722325 | 109230 | 74096 |
|  | CLOV280216-2_2 |  | *L. danicus* |  | 152510 | 107628 |
|  | CLOV280216-2_3 |  | *L. danicus* |  | 155134 | 111311 |
|  | CLOV280216-4_1 | 28 Feb 2016 | *L. danicus* | MH722326 | 149715 | 101470 |
|  | CLOV280216-4_2 |  | *L. danicus* |  | 193207 | 145823 |
|  | CLOV280216-4_3 |  | *L. danicus* |  | 169142 | 106947 |
|  | CLOV280216-7_1 | 28 Feb 2016 | *L. danicus* | MH722327 | 143447 | 104134 |
|  | CLOV280216-7_2 |  | *L. danicus* |  | 126757 | 93253 |
|  | CLOV280216-7_3 |  | *L. danicus* |  | 132608 | 95287 |
| **Coogee** | COOG100215-7_1 | 10 Feb 2015 | *L. aporus* | KY008568^#^ | 142460 | 67927 |
|  | COOG100215-7_2 |  | *L. aporus* |  | 145301 | 65118 |
|  | COOG100215-7_3 |  | *L. aporus* |  | 167721 | 79124 |
| **Maroubra** | MAR091215-5_1 | 9 Dec 2015 | *L. danicus* | MH722328 | 135455 | 96020 |
|  | MAR091215-5_2 |  | *L. danicus* |  | 160359 | 112947 |
|  | MAR091215-5_3 |  | *L. danicus* |  | 168873 | 88118 |
|  | MAR091215-6_1 | 9 Dec 2015 | *L. danicus* | MH722329 | 105339 | 78634 |
|  | MAR091215-6_2 |  | *L. danicus* |  | 157445 | 123195 |
|  | MAR091215-6_3 |  | *L. danicus* |  | 165570 | 128472 |
|  | MAR091215-9_1 | 9 Dec 2015 | *L. convexus* | KY008566^#^ | 157069 | 119279 |
|  | MAR091215-9_2 |  | *L. convexus* |  | 171708 | 125659 |
|  | MAR091215-9_3 |  | *L. convexus* |  | 159512 | 97530 |
|  | MAR091215-10_1 | 9 Dec 2015 | *L. convexus* | KY008565^#^ | 163549 | 126110 |
|  | MAR091215-10_2 |  | *L. convexus* |  | 135058 | 110723 |
|  | MAR091215-10_3 |  | *L. convexus* |  | 162246 | 122980 |
|  | MAR091215-11_1 | 9 Dec 2015 | *L. convexus* | KY008567^#^ | 174389 | 147651 |
|  | MAR091215-11_2 |  | *L. convexus* |  | 157874 | 127400 |
|  | MAR091215-11_3 |  | *L. convexus* |  | 210631 | 173004 |
|  | MAR091215-12_1 | 9 Dec 2015 | *L. danicus* | KY008553^#^ | 138983 | 100278 |
|  | MAR091215-12_2 |  | *L. danicus* |  | 153218 | 103015 |
|  | MAR091215-12_3 |  | *L. danicus* |  | 149666 | 105437 |
| **Twofold** | TF251016-1_1 | 25 Oct 2016 | *L. danicus* | MH722330 | 191948 | 140220 |
| **Bay** | TF251016-1_2 |  | *L. danicus* |  | 156002 | 121267 |
|  | TF251016-1_3 |  | *L. danicus* |  | 185164 | 143194 |
|  | TF251016-2_1 | 25 Oct 2016 | *L. danicus* | MH722331 | 128721 | 94934 |
|  | TF251016-2_2 |  | *L. danicus* |  | 156565 | 115272 |
|  | TF251016-2_3 |  | *L. danicus* |  | 155168 | 118969 |
|  | TF251016-4_1 | 25 Oct 2016 | *L. danicus* | MH722332 | 121041 | 85146 |
|  | TF251016-4_2 |  | *L. danicus* |  | 142690 | 92542 |
|  | TF251016-4_3 |  | *L. danicus* |  | 160612 | 127662 |
|  | TF251016-5_1 | 25 Oct 2016 | *L. danicus* | MH722333 | 147159 | 67787 |
|  | TF251016-5_2 |  | *L. danicus* |  | 160124 | 77013 |
|  | TF251016-5_3 |  | *L. danicus* |  | 152226 | 71020 |
|  |  |  |  |  |  |  |

* strain died before ITS confirmation; strain identified by morphology/presence of auxospores
