# Supplementary material for: The Microbiome of the Cosmopolitan Diatom Leptocylindrus Reveals Significant Spatial and Temporal Variability
Source: Front Microbiol. 2018 Nov 15;9:2758. doi: 10.3389/fmicb.2018.02758 (PMC6249420; doi:10.3389/fmicb.2018.02758)
Supplement: Supplementary file 5 [file Table_5.docx]

Supplementary Table 5. Analysis of similarity (ANOSIM) pair-wise tests (Bray-Curtis) and SIMPER results using bacterial relative abundance data [log (x + 1) transformed]; SIMPER analysis identified species with >5% cumulative contribution to dissimilarity among species and sampling locations (Sydney sites combined); bold taxa are higher in relative abundance at corresponding bold location; *p<0.05 significant; for OTU expanded names see Supplementary Table 1.

| **Pairwise Comparisons** | | **Bacterial Relative Abundance** | | |
| --- | --- | --- | --- | --- |
|  | R statistic | | *P*-value |  |
| **CH**, FOS | 0.793 | | 0.001* | **OTU_780 Bacteroidetes; Flavobacteriia; Flavobacteriales; Cryomorphaceae; Owenweeksia; uncultured bacterium**  **OTU_898 Bacteroidetes; Flavobacteriia; Flavobacteriales; Cryomorphaceae; Owenweeksia; uncultured bacterium**  **OTU_647 Proteobacteria; Gammaproteobacteria; Alteromonadales; Alteromonadaceae; Alteromonas; Ambiguous_taxa**  OTU_7700 Proteobacteria; Alphaproteobacteria; Rhodobacterales; Rhodobacteraceae; Roseovarius; Ambiguous_taxa  **OTU_10722 Proteobacteria; Alphaproteobacteria; Sphingomonadales; Erythrobacteraceae; Erythrobacter; uncultured bacterium** |
| CH, **SYD** | 0.76 | | 0.001* | **OTU_7767 Proteobacteria; Alphaproteobacteria; Rhodobacterales; Rhodobacteraceae; Roseovarius; uncultured bacterium**  OTU_647 Proteobacteria; Gammaproteobacteria; Alteromonadales; Alteromonadaceae; Alteromonas; Ambiguous_taxa  **OTU_9389 Proteobacteria; Alphaproteobacteria; Rhodobacterales; Rhodobacteraceae; Donghicola; Ambiguous_taxa**  OTU_8574 Proteobacteria; Alphaproteobacteria; Sneathiellales; Sneathiellaceae; Sneathiella; uncultured bacterium  OTU_780 Bacteroidetes; Flavobacteriia; Flavobacteriales; Cryomorphaceae; Owenweeksia; uncultured bacterium  OTU_10722 Proteobacteria; Alphaproteobacteria; Sphingomonadales; Erythrobacteraceae; Erythrobacter; uncultured bacterium |
| **CH**, TF | 0.987 | | 0.001* | OTU_432 Proteobacteria; Gammaproteobacteria; Alteromonadales; Pseudoalteromonadaceae; Pseudoalteromonas; uncultured bacterium  **OTU_898 Bacteroidetes; Flavobacteriia; Flavobacteriales; Cryomorphaceae; Owenweeksia; uncultured bacterium**  **OTU_780 Bacteroidetes; Flavobacteriia; Flavobacteriales; Cryomorphaceae; Owenweeksia; uncultured bacterium**  **OTU_9210 Proteobacteria; Alphaproteobacteria; Rhodobacterales; Rhodobacteraceae; Ambiguous_taxa; Ambiguous_taxa** |
| FOS, **SYD** | 0.48 | | 0.001* | **OTU_9389 Proteobacteria; Alphaproteobacteria; Rhodobacterales; Rhodobacteraceae; Donghicola; Ambiguous_taxa**  OTU_668 Bacteroidetes; Flavobacteriia; Flavobacteriales; Cryomorphaceae; Owenweeksia; Cryomorphaceae bacterium IMCC2672  OTU_7700 Proteobacteria; Alphaproteobacteria; Rhodobacterales; Rhodobacteraceae; Roseovarius; Ambiguous_taxa  **OTU_537 Bacteroidetes; Flavobacteriia; Flavobacteriales; Flavobacteriaceae; Tenacibaculum; Ambiguous_taxa**  OTU_10188 Proteobacteria; Alphaproteobacteria; Rhodobacterales; Rhodobacteraceae; Sulfitobacter; Ambiguous_taxa  **OTU_712 Proteobacteria; Gammaproteobacteria; Cellvibrionales; Halieaceae; Haliea; Ambiguous_taxa**  OTU_4777 Proteobacteria; Gammaproteobacteria; Cellvibrionales; Halieaceae; Parahaliea; uncultured Haliea sp.  **OTU_10830 Proteobacteria; Alphaproteobacteria; Rhodobacterales; Rhodobacteraceae; uncultured; uncultured bacterium** |
| **FOS**, TF | 0.774 | | 0.001* | **OTU_9210 Proteobacteria; Alphaproteobacteria; Rhodobacterales; Rhodobacteraceae; Ambiguous_taxa; Ambiguous_taxa**  OTU_432 Proteobacteria; Gammaproteobacteria; Alteromonadales; Pseudoalteromonadaceae; Pseudoalteromonas; uncultured bacterium  **OTU_7700 Proteobacteria; Alphaproteobacteria; Rhodobacterales; Rhodobacteraceae; Roseovarius; Ambiguous_taxa**  **OTU_668 Bacteroidetes; Flavobacteriia; Flavobacteriales; Cryomorphaceae; Owenweeksia; Cryomorphaceae bacterium IMCC2672**  **OTU_10188 Proteobacteria; Alphaproteobacteria; Rhodobacterales; Rhodobacteraceae; Sulfitobacter; Ambiguous_taxa** |
| **SYD**, TF | 0.648 | | 0.001* | OTU_432 Proteobacteria; Gammaproteobacteria; Alteromonadales; Pseudoalteromonadaceae; Pseudoalteromonas; uncultured bacterium  **OTU_9210 Proteobacteria; Alphaproteobacteria; Rhodobacterales; Rhodobacteraceae; Ambiguous_taxa; Ambiguous_taxa**  **OTU_9389 Proteobacteria; Alphaproteobacteria; Rhodobacterales; Rhodobacteraceae; Donghicola; Ambiguous_taxa**  **OTU_9213Proteobacteria; Alphaproteobacteria; Rhodobacterales; Rhodobacteraceae; Roseobacter clade NAC11-7 lineage**  **OTU_7767 Proteobacteria; Alphaproteobacteria; Rhodobacterales; Rhodobacteraceae; Roseovarius; uncultured bacterium** |
